# The Hospital World

**Published:** 1910-12-03

**Authors:** 


### The Hospital World.

### MR. WALTER SCOTT SETON-KARR.

Guy's Hospital loses a keen supporter and firm friend in Mr. Walter Scott Seton-Karr, who died last week at the age of eighty-eight years. He was elected in 1882 as a governor of the hospital, his colleague for the year being Mr. Cosmo Bonsor, who now holds the office of President. From the first Mr. Seton-Karr took a great interest in the institution. He regularly attended meetings of the court, visited the wards, and generally devoted much of his time and attention to the hospital. From the *Guy's Hospital Gazette* we take the following further particulars about his career :—

Mr. Seton-Karr was a son of Mr. Andrew Seton, the East India Company's commercial resident at Maldah, who, in 1815, inherited the estate of Kippilaw, near Abbottsford, and, in accordance with the entail provision, took the additional surname of Karr. Sir Walter Scott was on intimate terms with Mr. Andrew Seton-Karr, and took great interest in young Seton-Karr, who was his godson and namesake, and in after-life delighted to relate his reminiscences of the great novelist.

Mr. Walter Scott Seton-Karr was educated at Rugby (where he took part in the football match described in *Tom Brown's Schooldays*) and at Haileybury. He then entered the Indian Civil Service, and held the Under-Secretaryship in Bengal from 1847 to 1853. He presided over a Commission of Inquiry into the indigo riots in Bengal, which occurred a few years after the Mutiny. In 1862 he went to Calcutta as a Judge of the High Court. Before retiring from the public service in 1870, he spent two years as Foreign Secretary to the Government of India, and he was also Vice-Chancellor of the University of Calcutta.

During his years of retirement, besides his association with Guy's Hospital, Mr. Seton-Karr was a governor of Marlborough College, was for many years on the Chelsea Board of Guardians, and took an active part in other charitable and educational work. He was on three occasions unsuccessful by narrow margins as a Conservative candidate for Parliament. He was renowned as a shot and angler, and even in his later years went regularly to Scotland every autumn to practise his favourite sports.

### Personalia.

At a meeting of the Special Court of Governors of the Radcliffe Infirmary, Oxford, on November 22, Dr. D. S. Mackintosh, M.V.O., was appointed adviser for the construction of an out-patient and dispensary department, and of chemical and pathological laboratories.

Ancoats Hospital, Manchester, lost by death an old friend and supporter in the person of Mr. S. L. Helm. His connection with the institution began as far back as 1884, when first he became a member of the Board. The simplest proof of the value of his work and the regard which his colleagues felt for him is to be found in his election in 1896 to the position of chairman, in which capacity he had even fuller scope for his interest in the hospital. It was Mr. Charles Behrens, the Lord Mayor of Manchester, who recalled these facts at the recent annual meeting, and every friend of Ancoats Hospital will be glad of this public witness to the services of its late chairman, who did good work for his institution in his day.

The Edinburgh Royal Infirmary has lost its superintendent by the resignation of Col. W. P. Warburton, M.D., C.S.I., who has held the post, we understand, for eleven years. Col. Warburton was in the Indian Medical Service from 1866 till his retirement in 1899, and among other posts had held that of Inspector-General of Civil Hospitals in the North-West Provinces. Commenting on his retirement the *British Medical Journal* says: "There are many deeply interested in the well-being of the Edinburgh Royal Infirmary who hope that the tradition of appointing as superintendent a man who has retired at the age limit, or on approaching that limit, from the Indian Medical or Army Medical Service, will now be broken. While holding strongly that the office should be held by a medical man, they think that the superintendent of a vast hospital like the Royal Infirmary should be a younger man, thoroughly equipped in all the details of modern medicine and surgery, and of wide mental outlook. The new appointment will be scrutinised more closely than ever before."

Mr. Elfred Chambers Austin, whom the Guardians have selected as medical superintendent of the Hope Hospital, Salford, entered upon his medical career at St. Mary's Hospital Medical School, Paddington, where he was a distinguished student. He qualified M.R.C.S., L.R.C.P., in 1899, and was then appointed resident obstetric officer to Dr. Handfield Jones and Dr. W. J. Gow at the hospital, which post he filled for six months. Later in the same year he was appointed house physician to the Leicester Infirmary. The senior resident posts in the large provincial hospitals were at that period eagerly sought after, and Mr. Austin was appointed to the Leicester Infirmary from a number of applicants. The post then carried with it duties of an administrative character, and at the close of Dr. Austin's year of office his work was most satisfactorily reported on by the honorary physicians. Subsequently Mr. Austin filled the post of resident medical officer to the Kensington Infirmary, and took a voyage as ship's surgeon. He was in 1903 admitted a Fellow of the Royal College of Surgeons of Edinburgh. Having obtained a large and varied experience of institutional work and of the various branches of his profession, Mr. Austin entered upon a partnership practice at Broughton Astley, near Leicester, where his work and experience quickly gained for him more than local reputation as a family physician. He was appointed one of the medical officers and public vaccinator of the Lutterworth Union, certifying factory surgeon, St. John Ambulance lecturer, as well as surgeon to the several local friendly societies. He was a member of the Leicester and Rutland branch of the British Medical Association, and contributed from time to time to the discussions on matters of professional interest. He was held in esteem by his brother practitioners in the district. Mr. Austin took a prominent part in the social life of the district in which he resided, and his services were frequently in demand to preside over local gatherings.

### Elections and Appointments.

The Duke of Devonshire has been re-elected President of the Derbyshire Royal Infirmary, and the following nominations of Governors to serve on the Weekly Board for the year were accepted: Sir Edwin Ann (Derby), Mr. Matthew (Derby), Mr. Arthur Cox (Derby), Mr. Henry Evans (Borrowash), Sir Arthur Heywood, Bart., Duffield Bank, Mr. Robert Knowles, Ednaston Lodge, Mr. William Malin (Derby), Mr. J. B. Marsden-Smedley, Lea Green (Cromford), Mr. G. W. Peach, Langley Hall (Derby), Mr. Geo. Herbert Strutt, Makeney (Derby), and Mr. A. Leslie Wright, Butterley Hall.

